# Combined Use of Anisotropic Silver Nanoprisms with Different Aspect Ratios for Multi-Mode Plasmon-Exciton Coupling

**DOI:** 10.1186/s11671-020-3248-8

**Published:** 2020-01-16

**Authors:** Naoto Takeshima, Kosuke Sugawa, Hironobu Tahara, Shota Jin, Masaki Noguchi, Yutaro Hayakawa, Yuhei Yamakawa, Joe Otsuki

**Affiliations:** 1 Department of Materials and Applied Chemistry, College of Science and Technology, Nihon University, Kanda-Surugadai, Chiyoda-ku, Tokyo, 101-8308 Japan; 2 Graduate School of Engineering, Nagasaki University, Bunkyo, Nagasaki, 852-8521 Japan

**Keywords:** Localized surface plasmon, Tetraphenyl porphyrin, Fluorescence enhancement, Absorption enhancement, Silver nanoprisms, Plasmon-exciton interaction

## Abstract

Absorption enhancement based on interaction between the localized surface plasmon (LSP) and molecular exciton is one of the most important phenomena for the development of high-performance solar devices. In this study, hybrids of plasmonic metal nanoparticles and dye molecules have been developed, which exhibit enhanced absorption at precisely tuned wavelengths in a visible region. The hybrids consist of a porphyrin derivative, which has four absorption peaks (Q-bands) in a range of 500–700 nm, and triangular silver nanoprisms (AgPRs), which are developed by us to exhibit precisely tuned LSP resonance wavelengths. Absorption enhancement over the whole Q-band range is induced by the combined use of three kinds of AgPRs of different aspect ratios. Furthermore, the quantitative evaluation of absorption enhancement based on the LSP-based fluorescence enhancement phenomenon has demonstrated that efficient absorption enhancement can be effected at multiple wavelengths.

## Introduction

Controlling light-matter interaction is one of the intensively researched topics in the photochemical science [1]. The utilization of metal nanoparticles has been recognized as a way to strengthen the light-matter interaction because they generate strong electromagnetic fields at the nanoscale dimension upon excitation of the localized surface plasmon (LSP) resonance. In particular, the interaction between LSP and exciton of photofunctional molecules is attracting much attention because of the occurrence of various beneficial optical phenomena including a huge emission enhancement [2], suppression of photochemical quenching [3], plasmonic resonance energy transfer [4], enhanced water splitting [5], and so on. Antosiewicz et al. classified the interactions between metal nanoparticles and photofunctional molecules into three regimes according to the strength of interaction: (1) the enhanced absorption regime, (2) the induced transparency regime, and (3) the strong coupling regime [6]. The strong coupling leads to the splitting of state in which the LSP and molecular exciton states are mixed, which is manifested in the splitting of the extinction peak at the wavelength of the molecular resonance. On the other hand, in the enhanced absorption regime, the absorption and scattering components of the LSP are damped, whereas the molecular absorption is increased by the energy transfer from the LSP to the molecule. The enhanced molecular absorption cancels the damped absorption component of LSP resonance but the uncompensated damped scattering component remains as a dip in the extinction spectrum. The induced transparency regime refers to an intermediate case between the absorption enhancement regime and the strong coupling regime. Among these, the absorption enhancement is quite important for the development of highly efficient solar devices [7–11]. Particularly, absorption enhancement over a wide range of wavelength is essential to utilize the wide solar spectrum. However, the multiwavelength generation of enhanced absorption through the interaction between the LSP and the exciton has never been demonstrated. The generation of spectral dips at multiple wavelengths was reported, which was achieved by using plasmonic metal nanoparticles in combination with two different dye molecules, but this phenomenon was attributed to the Rabi oscillation in the strong coupling case and did not lead to absorption enhancement [12]. In another report on the plasmon-exciton coupling at multiple wavelengths using a combination of cyanine dye molecules exhibiting two absorption bands and metal nanorods [13], the absorption enhancement was not identified.

In this study, we have succeeded in enhancing light absorption at multiple wavelengths over a visible region through the interaction between the exciton of dye molecules and the LSP resonances of three different kinds of plasmonic metal nanoparticles. The achievement was made possible by developing a precise tuning technique of LSP wavelength of triangular silver nanoprisms (AgPRs). Furthermore, the enhancement factors of absorption were quantitatively evaluated utilizing the fluorescence enhancement, which occurred as a result of the interaction of exciton of dye molecules with the LSP resonance.

## Method/Experimental

### Materials

Milli-Q-grade water (resistivity: 18.2 MΩ cm) was used for the preparation of all aqueous solutions. Toluene was purchased from Kishida Chemical (Japan). 5,10,15,20-Tetraphenyl-21H,23H-porphyrin (TPP), sodium tetrahydroborate (NaBH_4_), silver nitrate (AgNO_3_), and polyethyleneimine (PEI) (MW ~ 10,000) were purchased from Fujifilm Wako Pure Chemical (Japan). Trisodium citrate dihydrate, sodium hydroxide (NaOH), ammonium solution (NH_3,_ 28%), and hydrogen peroxide solution (H_2_O_2_, 30%) were purchased from Kanto Chemical (Japan). All chemicals were used without further purification.

### Measurements

Transmission electron microscopy (TEM) and atomic force microscopy (AFM, tapping mode) were carried out using a Hitachi HF-2000 microscope and a Hitachi SPI-3800N-SPA400 microscope, respectively. The extinction spectra of sample substrates were measured by a normal transmittance setup using a JASCO V-770 spectrometer. The absorption and scattering spectra were measured by the spectrometer (JASCO V-770) equipped with an integrating sphere according to a previous report [14]. Fluorescence excitation spectra of sample substrates were measured by a JASCO FP-8600 fluorescence spectrophotometer. The calculation for the extinction spectra of AgPRs was conducted using the boundary element method (BEM) with retarded electromagnetic fields for the full Maxwell equations [15]. To produce the random orientation of the AgPRs in solution phase, the transmission spectra were averaged over all allowed combinations of incident light polarization (*E*_*x*_, *E*_*y*_, *E*_*z*_) and incident light propagation orientations (*k*_*x*_, *k*_*y*_, *k*_*z*_). The geometry models are shown in Additional file 1: Figure S1. The dielectric function of silver was taken from the previous report by Rakic et al. [16].

### Synthesis of AgPRs with Different Resonance Wavelengths

The AgPRs with precisely tuned resonance wavelengths of the in-plane dipole mode (500, 540, 560, 625, 645, and 675 nm) were synthesized by the light-mediated method developed by us. An aqueous solution (100 mL) containing trisodium citrate (5 mM) as a protective agent and NaBH_4_ (0.2 mM) as a reducing agent was injected into an aqueous solution (100 mL) of AgNO_3_ (1 mM) in an ice bath under stirring. The mixture was further stirred for 1 h, leading to the formation of Ag nanospheres with an average diameter of 11 nm. After an aqueous solution of NaOH (0.2 M, 100 μL) was injected into the colloidal solution of Ag nanospheres (10 mL) to adjust the pH to 11.2, light-emitting diode (LED) light was irradiated, which led to the formation of AgPRs. Specifically, the resonance wavelength of in-plane dipole mode of AgPRs was precisely adjusted by sequential irradiation of LED light with different wavelengths of 470 ± 5 nm (5800 mcd, 3 × 3 arrays), 525 ± 5 nm (18,000 mcd, 3 × 3 arrays), and 590 ± 5 nm (50,000 mcd, 3 × 3 arrays) for predetermined periods of time as summarized in Table 1, whereas the irradiation setup is shown in Additional file 1: Figure S2. The obtained AgPRs are designated as AgPRs-*X*, in which *X* indicates the resonance wavelength.

**Table 1:** Synthetic conditions for AgPRs with various resonance wavelengths

1: Irradiation period of 470 nm/h	2: Irradiation period of 525 nm/h	3: Irradiation period of 590 nm/h	Resonance wavelength^a^ of AgPRs/nm
24	0	0	500 ± 2.0
4	20	0	540 ± 1.2
2	22	0	560 ± 0.6
0	6	42	625 ± 3.5
0	3	45	645 ± 5.0
0	2	46	675 ± 1.7

^a^Mean values and standard deviations

### Preparation of Hybrids of TPP and AgPRs

To clean the surface, a glass substrate (1.5 × 2.0 cm^2^) was immersed into a mixed aqueous solution of 30% H_2_O_2_ and 28% NH_3_ (1/1 = V/V) at 100 °C for 3 h, followed by washing with Milli-Q water. The cleaned substrate was kept in Milli-Q water until use. The substrate was modified with positively-charged PEI by immersing it into an aqueous solution of PEI (4.2 mg/mL) for 1 min, followed by washing with Milli-Q water. The positively charged substrate was then immersed in the colloidal solution to electrostatically immobilize AgPRs, which were negatively charged owing to the citric acid sheath. The immersion was continued until the extinction intensity of the main LSP resonance band (in-plane dipole mode) reached 0.2; thus obtained substrate is referred to as AgPRs-*X*/glass. The extinction intensity of the LSP of AgPRs on the glass plate was set to be 0.2 to avoid the LSP coupling between adjacent AgPRs observed for denser samples [17]. For the preparation of a glass plate on which three AgPRs (LSP resonance wavelengths of 500, 560, and 645 nm) were immobilized (AgPRs-ternary/glass), the positively charged glass plate was immersed sequentially into respective colloidal solutions of AgPRs until each extinction intensity of the main LSP resonance reached 0.1. A toluene solution of TPP (1.5 mM) was spin-coated (3000 rpm, 30 s) on AgPRs-*X*/glass and AgPRs-ternary/glass, affording the hybrids of TPP and AgPRs (TPP/AgPRs-*X* and TPP/AgPRs-ternary). TPP was also deposited on a bare glass substrate as a reference by spin-coating the TPP solution onto the PEI-modified glass substrate (TPP/glass).

## Results and Discussion

### Optical Properties of TPP and Optical Properties and Morphologies of AgPRs

**Fig. 1:**
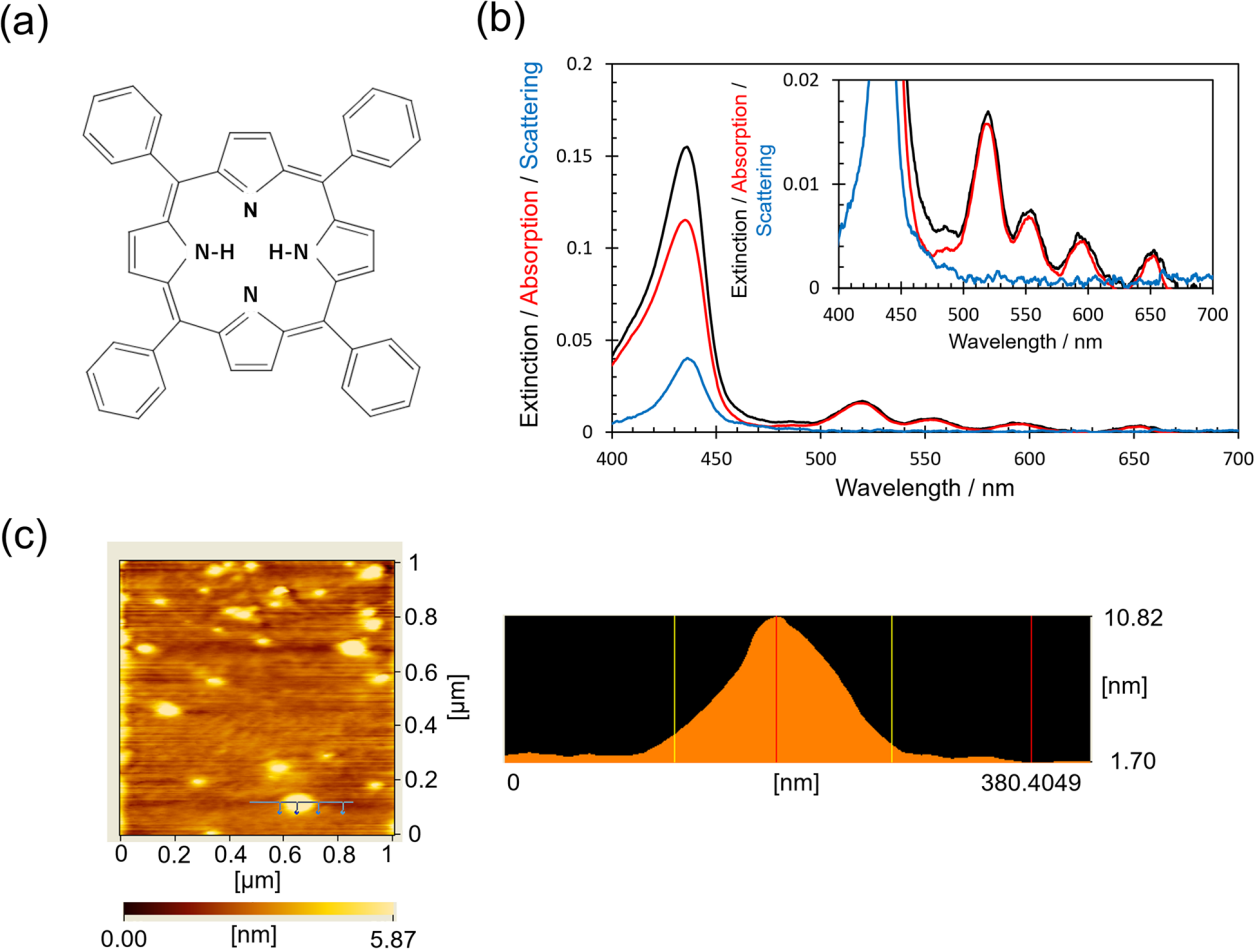
**a** Molecular structure of TPP. **b** Extinction (black line), absorption (red line), and scattering (blue line) spectra of TPP/glass. Inset shows magnification of Q-bands. **c** AFM image of the surface of TPP/glass

In this study, a porphyrin derivative, TPP (molecular structure: Fig. 1a), was employed as a photofunctional molecule. Porphyrins, synthetic analogues of natural chlorophylls, are frequently used as light harvester owing to their wide absorption in the visible region [18]. However, the absorption coefficients of four absorption peaks in 500–700 nm region are relatively low (Q-bands, absorption coefficient: ~ 10^4^ M^−1^ cm^−1^), whereas the absorption around 420 nm is quite strong (Soret band, absorption coefficient: > 10^5^ M^−1^ cm^−1^). Therefore, we set out an attempt to enhance the absorption of Q-bands by means of the LSP of metal nanoparticles. Figure 1b shows the extinction, absorption, and scattering spectra of TPP/glass. An extinction peak of the Soret band at 435 nm and four extinction peaks of the Q-bands at 519, 552, 596, and 653 nm [19] were observed. Compared to the peak wavelengths of Q-bands in a toluene solution of TPP (514, 548, 591, and 649 nm, Additional file 1: Figure S3), those of the TPP/glass were slightly red-shifted. Also, the extinction at the Soret band was accompanied by a prominent scattering component. These results suggest that the TPP molecules densely aggregated on the glass substrate because the scattering cross section of molecular aggregates is proportional to the square of the volume of the aggregates and the red-shift can be attributed to the π-π interaction of TPP [20]. To investigate the morphology of the molecular aggregates, the AFM measurement was conducted for TPP/glass. As shown in Fig. 1c, the glass surface was scattered with the molecular aggregates having a height of 7 ± 2 nm and a diameter of 108 ± 29 nm.

A large-scale, reproducible synthesis of plasmonic metal nanoparticles which generate strong LSP resonance at precisely tuned wavelengths is highly desired for a large-scale applications. Absorption enhancement of the Q-bands of porphyrins spread over a macroscopic substrate is a model case for such applications, which we will describe in this work. A satisfactory technique of metal nanoparticle preparation that meets the demand has been rarely reported [21, 22].

**Fig. 2:**
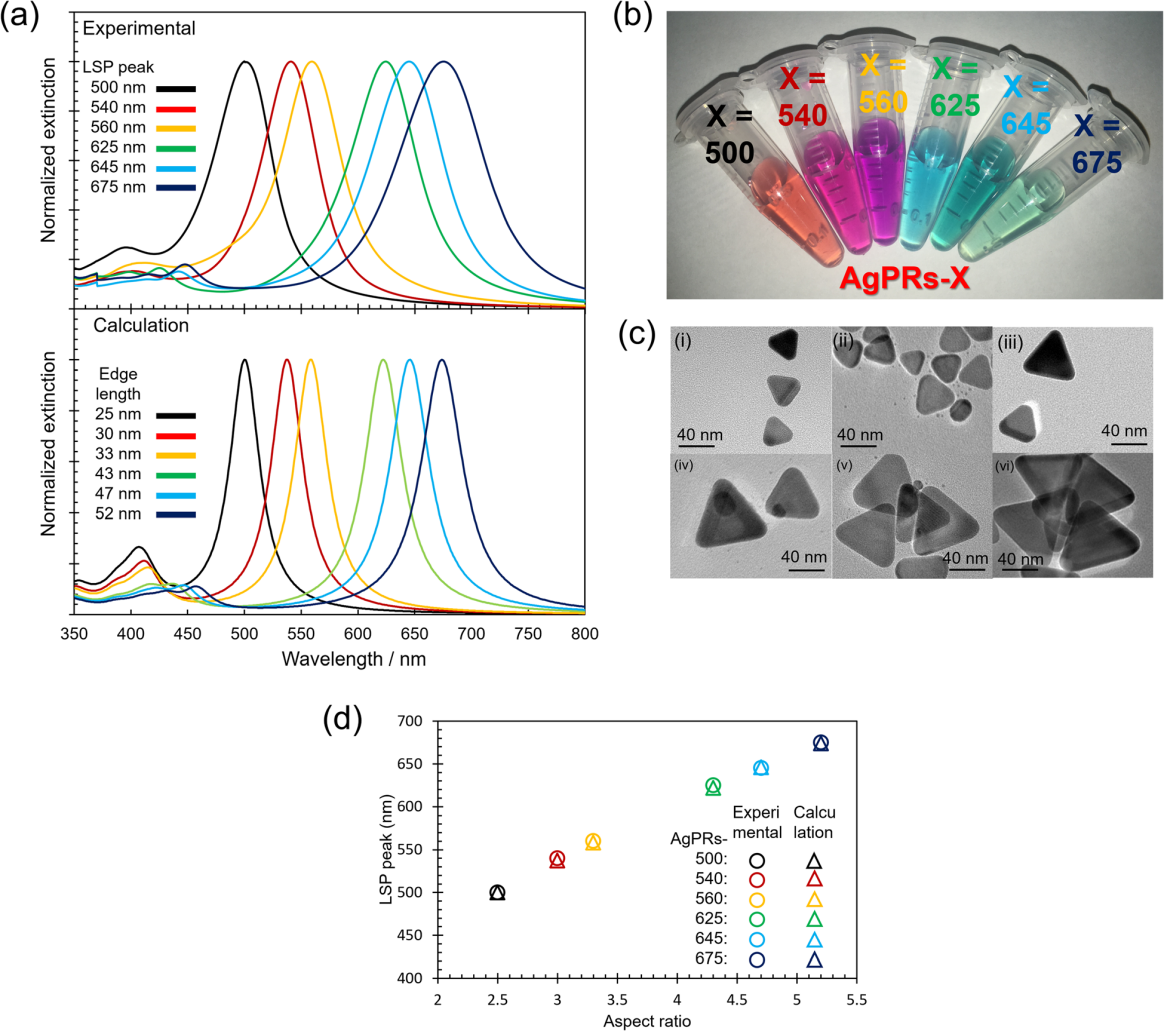
Characterization of optical property and morphology of AgPRs. **a** Normalized extinction spectra of aqueous solutions of AgPRs with different LSP peak wavelengths, and normalized extinction spectra calculated by BEM. **b** Photo image of synthesized AgPRs. **c** TEM images of various AgPRs (i-vi) synthesized by the modified photochemical methods. **d** Plots of LSP peaks of AgPRs against their aspect ratios

We have succeeded in synthesizing AgPRs which can generate strong local electromagnetic fields at precisely tuned LSP resonance wavelengths in sufficient amount [17, 23]. The wavelength region in which the LSP resonance occurs matches the Q-band region of porphyrins. Our method is based on the light-mediated method, which was originally developed by Mirkin’s research group [24, 25]. In the preparation, the AgPRs are synthesized by the irradiation of light to citrate-stabilized Ag nanospheres with a diameter below 10 nm. Hot electrons and holes are formed during the decay of the LSP resonance upon light irradiation. While the hot holes are transferred to citric acid adsorbed on the Ag surfaces, the hot electrons reduce silver ions, resulting in the formation of AgPRs. Although the LSP resonance wavelength was controlled by choosing the excitation light wavelength to a certain degree, the precise tuning has never been achieved by a single excitation wavelength in the previous reports [25–27]. In this study, we have succeeded in preparing AgPRs exhibiting the LSP resonance wavelengths with an unprecedented precision. This was achieved by tuning the sequence, wavelengths, and duration of irradiation (see experimental section and Table 1) in the process of converting the Ag nanospheres into AgPRs. For example, irradiation of 470 nm light alone produced AgPRs exhibiting the LSP resonance at 500 nm. Irradiation of 470 nm light followed by 525 nm light (keeping the total irradiation time unchanged) produced AgPRs with a red-shifted LSP resonance. During the first irradiation process of the 470 nm light, small AgPRs were formed by oriented attachment-like two-dimensional coalescence of Ag nanospheres. In the second 525 nm light irradiation, the AgPRs grew in an Ostwald ripening process to a specific size at the consumption of the remaining Ag nanospheres. Thus, obtained AgPRs generated the LSP resonance at exact wavelengths of 500, 540, 560, 625, 645, and 675 nm with small standard deviations (0.6–5 nm, see Table 1) under our specific conditions. The extinction spectra of respective colloidal solutions of AgPRs prepared five times are shown in Additional file 1: Figure S4, which clearly indicates that our synthetic method has a remarkable reproducibility in generating LSP resonances at exact wavelengths. Normalized extinction spectra and a photograph of the colloidal aqueous solutions of the obtained AgPRs are shown in Fig. 2a, b, respectively. All the AgPRs showed a prominent resonance band within 500–700 nm. It was found by comparing the LSP wavelengths and the TEM images, which are shown in Fig. 2c, that the resonance band was red-shifted with increasing the edge length (AgPRs-500: 25 ± 3 nm, AgPRs-540: 30 ± 4 nm, AgPRs-560: 33 ± 5 nm, AgPRs-625: 44 ± 9 nm, AgPRs-645: 47 ± 10 nm, and AgPRs-675: 52 ± 7 nm). Since the thicknesses of AgPRs which are synthesized by the photochemical method are almost constant at *ca.* 10 nm regardless of their edge lengths [28], the difference in the resonance wavelengths can be attributed to the difference in the aspect ratio (the ratio of edge length to thickness) [29]. To prove the correlation between the LSP wavelength and its aspect ratio, we calculated the extinction spectra using the BEM for AgPRs having the experimentally obtained edge lengths and a fixed thickness of 10 nm which were surrounded by aqueous phase (refractive index: 1.333) (Fig. 2a). The calculated resonance wavelengths agreed very well with the experimentally obtained wavelengths (Fig. 2d), which indicated that the accurate control of resonance band of AgPRs was achieved by precisely controlling the aspect ratio. The experimental extinction spectra of AgPRs were somewhat broader than the calculated ones. This may be partly because there was a distribution in the aspect ratios, albeit narrow, in the prepared AgPRs and partly because the solvent molecules (water) caused the chemical interface damping [30], both of which were not included in the calculations.

### Interaction Between LSP of AgPRs and Exciton of TPP

**Fig. 3:**
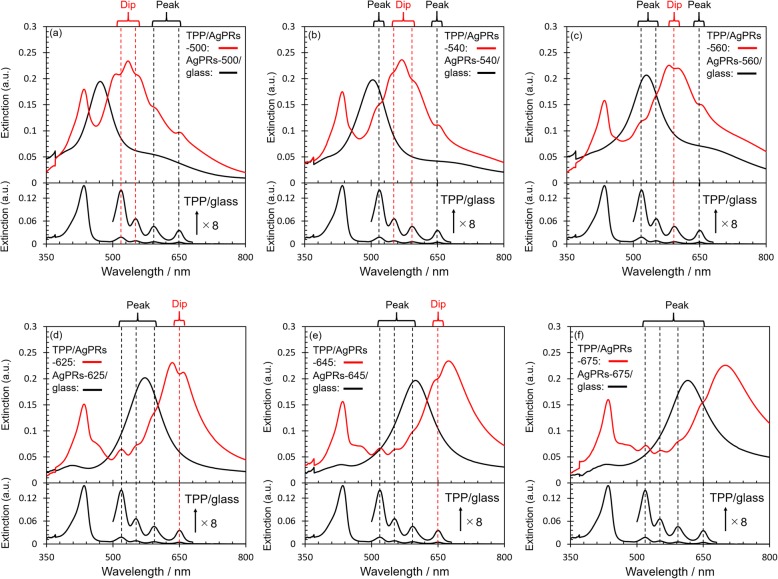
Extinction spectra. Dashed lines on the spectra represent absorption peak wavelengths of Q-bands of TPP/glass. **a** AgPRs-500/glass. **b** AgPRs-540/glass. **c** AgPRs-560/glass. **d** AgPRs-625/glass. **e** AgPRs-645/glass. **f** AgPRs-675/glass

To evaluate the interaction between the LSP of AgPRs and the exciton of TPP in TPP/AgPRs-*X*, the extinction spectra were measured for TPP/glass (as a reference), AgPRs/glass, and TPP/AgPRs-*X* (Fig. 3). The dashed lines in Fig. 3 indicate the peak wavelengths of Q-bands observed on TPP/glass. The resonance wavelengths of the in-plane dipole mode for all AgPRs/glass were blue-shifted by several tens of nanometers as compared with those in an aqueous phase (Fig. 2a). These shifts are attributed to a change in the refractive index of the medium surrounding the AgPRs from the aqueous phase to air (refractive index: 1.000) [31–33]. After spin-coating the TPP solution onto AgPRs-*X*/glass, the Soret band was observed at 436 nm. In addition, the LSP resonance bands were red-shifted, resulting in the LSP band within 500–700 nm for all AgPRs. These results suggest that the AgPRs were covered with TPP aggregates because the refractive index (approximately 1.6) of TPP is larger than that of air [18]. Note that prominent peaks or dips were observed at the wavelengths of the Q-band peaks on the LSP resonance band for all TPP/AgPRs-*X*. For example, in the case of TPP/AgPRs-500, while spectral dips were observed at 515 and 552 nm where the LSP resonance was strongly excited, peaks were observed at 595 and 654 nm in the edge region of the LSP resonance band. The latter positions are in the peripheral region of the LSP band where the electromagnetic field around the AgPRs is weak. Therefore, the coupling between the LSP resonance and the TPP exciton is weak, resulting in the overall spectrum resembling the sum of individual spectra. On the other hand, only peaks were observed for TPP/AgPRs-675 because the Q-bands overlap only with the peripheral region of the LSP band (Fig. 3f), suggesting inefficient interaction between the LSP and the exciton [34]. We emphasize based on these data that the strong interaction between the LSP and exciton, which is manifested by the appearance of dips, is efficiently induced only at a narrow wavelength region where the LSP resonance is strongly excited. Therefore, the combined use of multiple AgPRs with the LSP resonance at multiple wavelengths over the Q-band region is required.

**Fig. 4:**
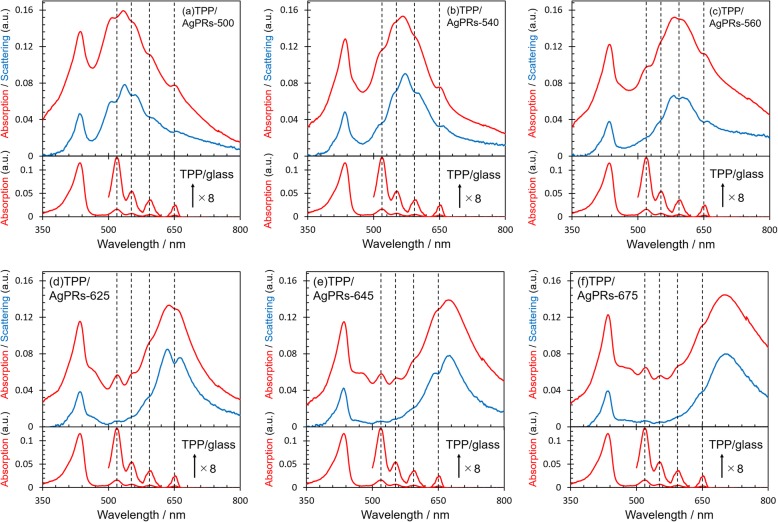
Absorption (red line) and scattering (blue line) spectra. Dashed lines on the spectra represent absorption peak wavelengths of Q-bands of TPP/glass. **a** TPP/AgPRs-500. **b** TPP/AgPRs-540. **c** TPP/AgPRs-560. **d** TPP/AgPRs-625. **e** TPP/AgPRs-645. **f** TPP/AgPRs-675

In the case classified as the absorption enhancement, which represents a weak coupling case, light absorption attributed to the exciton generation increases, whereas the extinction of LSP at the same wavelength decreases. As a result, the total absorption component changes little because the absorption enhancement to generate excitons is canceled out by the absorption decrease in the LSP band. On the other hand, the net scattering component decreases, resulting in a dip in the total extinction spectrum [6]. In the case of the strong coupling, prominent dips are observed similarly in both absorption and scattering spectra because two hybrid states separated in energy are formed in place of independent eigenstates. The induced transparency refers an intermediate case between the absorption enhancement and the strong coupling [6, 35, 36]. To further clarify the strength of interaction between the LSP and the exciton in our hybrids, the absorption and scattering spectra of TPP/AgPRs-*X* were measured (Fig. 4) [6, 37, 38]. Although prominent dips were observed in the region where the LSP was strongly excited in the scattering spectra for all TPP/AgPRs-*X* except for TPP/AgPRs-675, the dips were less prominent in the corresponding absorption spectra. These observations suggested that our hybrids TPP/AgPRs-500, 540, 560, 625, and 645 were in the regime of absorption enhancement with respect to the coupling strength.

### Realization of Enhanced Absorption Over the Whole Q-Bands

**Fig. 5:**
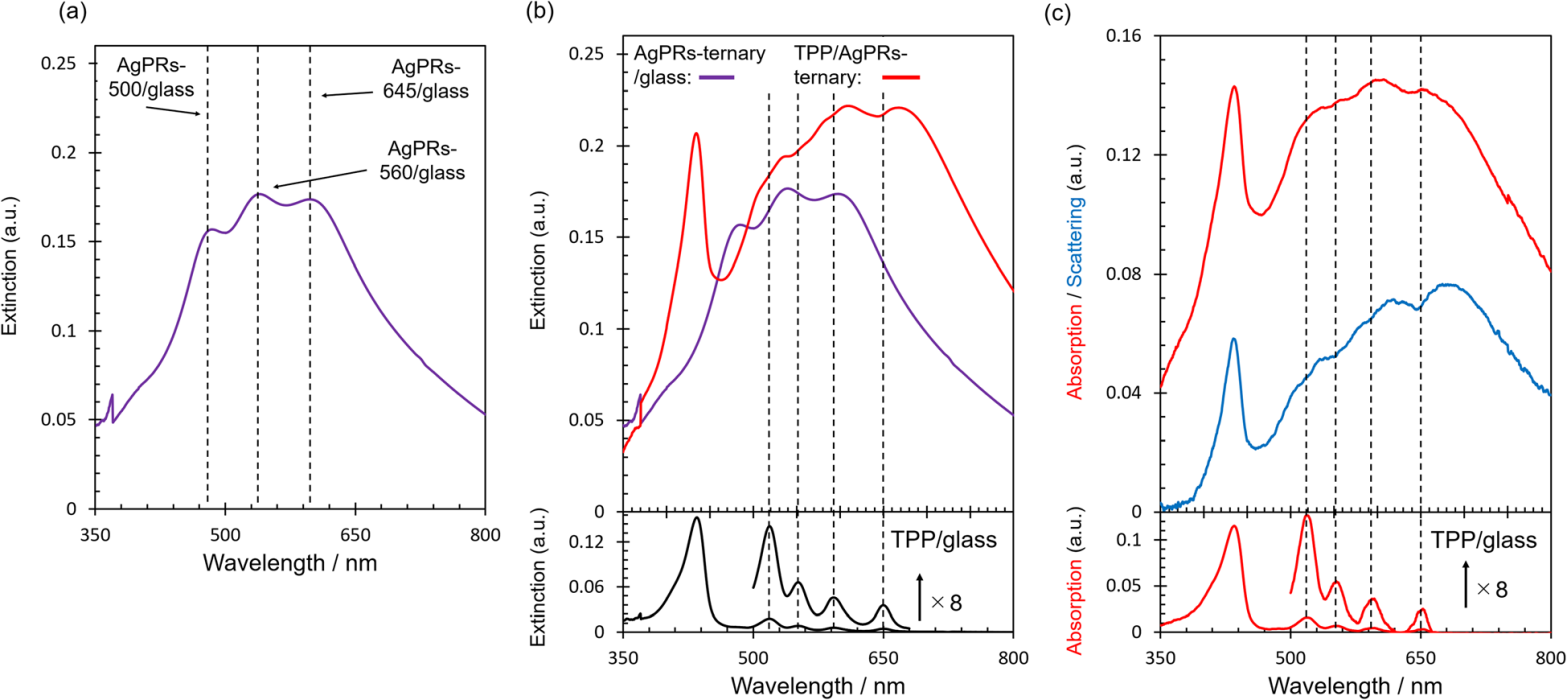
**a** Extinction spectra of AgPRs-ternary/glass. Dashed lines represent LSP peaks of AgPRs-500/glass, AgPRs-560/glass, and AgPRs-645/glass. **b** Extinction spectra of AgPRs-ternary/glass, TPP/AgPRs-ternary, and TPP/glass. **c** Absorption and scattering spectra of TPP/AgPRs-ternary and TPP/glass

Although we have succeeded in achieving the absorption enhancement at the region where the LSP resonance is strongly excited, absorption enhancement over a wider range covering the whole Q-bands may be beneficial in terms of solar light utilization. To achieve this, we hybridized TPP and AgPRs-ternary/glass (denoted as TPP/AgPRs-ternary). The extinction spectrum of AgPRs-ternary/glass is shown in Fig. 5a. Three distinct bands were observed at 485, 540, and 598 nm, which were assigned to the LSP resonance bands of AgPRs-500, 560, and 645, respectively. The extinction spectrum of TPP/AgPRs-ternary, which is shown in Fig. 5b, exhibited four dips at the wavelengths corresponding to the Q-band peaks. Furthermore, as shown in Fig. 5c, while the prominent four dips were observed at the Q-band wavelengths in the scattering spectrum, these dips did not appear in the absorption spectrum. These results suggested that the strength of interaction between the LSPs of AgPRs-500, 560, and 645 and the excitons generated in the whole Q-band wavelengths was in the enhanced absorption regime.

### Effect of Absorption Enhancement on Photodynamics of TPP

**Fig. 6:**
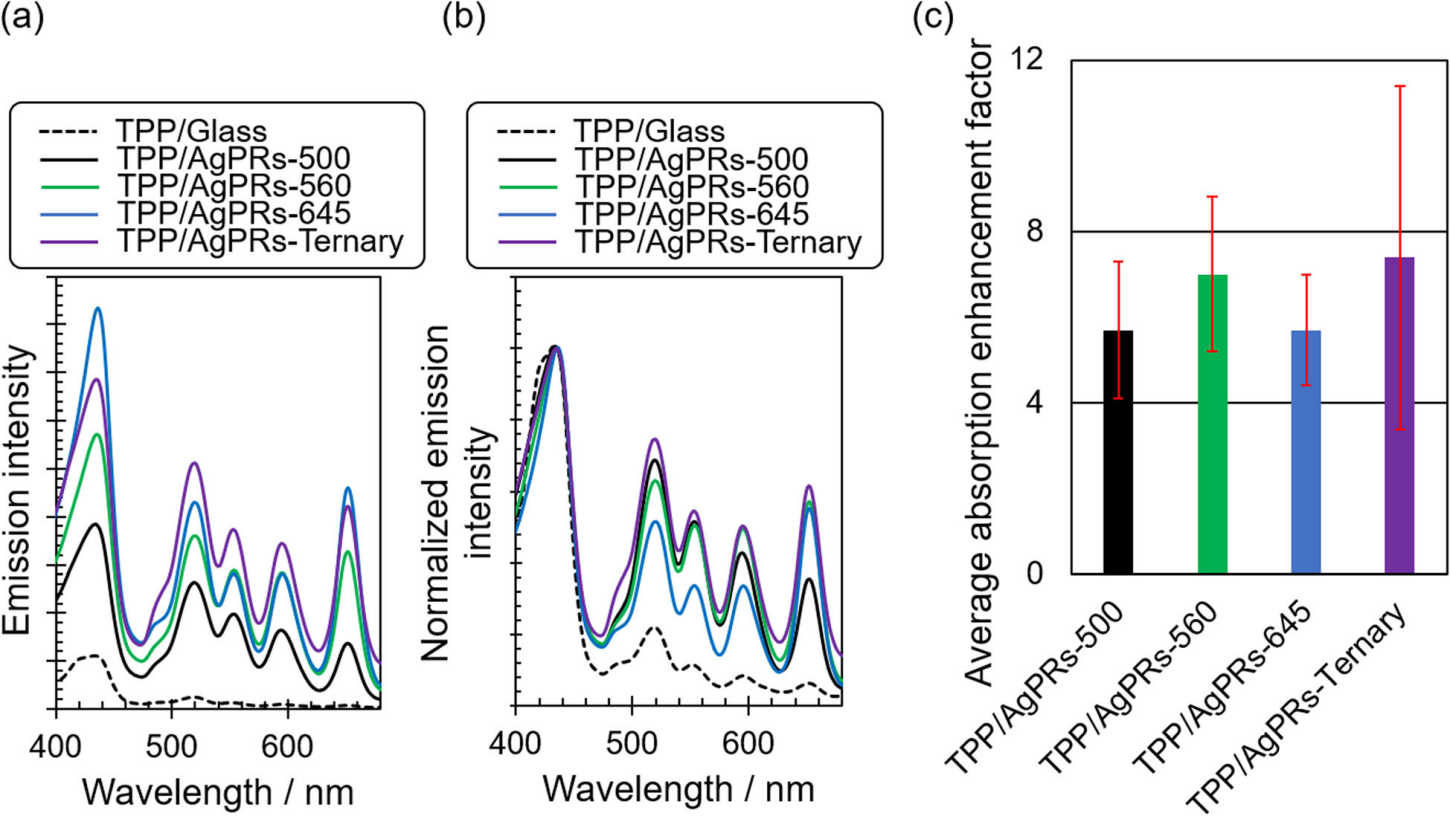
**a** Fluorescence excitation spectra as measured. **b** Normalized fluorescence excitation spectra (*λ*_em_ = 720 nm). **c** Average absorption enhancement factors of TPP/glass, TPP/AgPRs-500, TPP/AgPRs-560, TPP/AgPRs-645, and TPP/AgPRs-ternary. The red lines indicate the standard deviations for the measurements repeated three times

To quantitatively investigate the effect of the absorption enhancement on the photodynamics of TPP, the fluorescence excitation spectra of TPP/AgPRs-500, 560, 645, and ternary (*λ*_em_ = 720 nm) were measured (Fig. 6a). The fluorescence radiation of these hybrids was significantly enhanced by excitation in the region of both the Soret and Q-bands as compared to that of TPP/glass. The fluorescence enhancement factors at the Q-band peaks were in the range of 11–71 (see Additional file 1: Figure S5a). The fluorescence enhancement owing to the LSP resonance can be attributed to two mechanisms: the photoexcitation enhancement (i.e., absorption enhancement), which is induced when the LSP resonance band overlaps with the photoexcitation wavelength, and the acceleration in radiative decay rate, which is induced when the LSP resonance band overlaps with the fluorescence wavelength. The fluorescence enhancement for the Q-bands excitation could be induced by both of these mechanisms because the photoexcitation and fluorescence wavelengths overlap with the LSP resonance bands of TPP/AgPRs-500, 560, 645, and ternary. On the other hand, the fluorescence was also enhanced by 2.9–6.4 times for the Soret band excitation (see Additional file 1: Figure S5a). It is likely that the enhancement is solely attributed to the acceleration in the radiative decay rate because the excitation wavelength is separated far from the main LSP resonance bands of AgPRs in this case. Thus, the fluorescence enhancement factors were calculated using the fluorescence excitation spectra normalized at the Soret band (435 nm, Fig. 6b), which can be attributed to the net absorption enhancement. The average enhancement factors for the Q-bands are shown in Fig. 6c, obtained by averaging enhancement factors for respective Q-band peaks (Additional file 1: Figure S5b). Consequently, TPP/AgPRs-ternary showed the absorption enhancement uniformly at all the Q-band peaks resulting in the enhancement factor of 7.4. This result indicated that the absorption enhancement based on plasmon-exciton coupling was achieved over the broad wavelength region by combined use of AgPRs with different aspect ratios, which points to the usefulness of our precise tuning technique of LSP wavelengths. Randomly distributed polydisperse AgPRs would also enhance a wide range of absorption but many molecules would be placed out of resonance with AgPRs. Combining AgPRs of which the LSP resonance wavelength is precisely tuned to the molecular absorption peak positions would be the most efficient strategy in harvesting a spectrum of light. Thus, our precise tuning technique is promising for the development of high-performance solar devices.

## Conclusion

We have succeeded in synthesizing AgPRs with well-defined resonance wavelengths, which can be precisely tuned over a wide visible region. The difference in resonance wavelengths is attributed to the difference in their aspect ratios. The combined use of AgPRs with three different aspect ratios led to the absorption enhancement over the whole Q-bands, which was demonstrated by their extinction, absorption, and scattering spectra. In addition, absorption enhancement factors were quantitatively evaluated from the fluorescence excitation spectra, which demonstrated the usefulness of our protocol to produce AgPRs exhibiting precisely tuned LSP resonance wavelengths in realizing absorption enhancement over the wide visible wavelength region. Thus, the combined use of AgPRs with different aspect ratios have a great potential for enhancing light-matter interaction in a wide wavelength region, which paves the way for the fabrication of high-performance optoelectronic devices including solar cells, photocatalysts, and bio-imaging sensors.

## Supplementary information

**Additional file 1: Figure S1.** Geometric models of AgPRs with various LSP resonance wavelengths. **Figure S2.** A Setup for the irradiation of LED light to a colloidal aqueous solution of Ag nanospheres. **Figure S3.** Normalized extinction and emission spectra of a toluene solution of TPP (1×10^-6^ M). **Figure S4.** Extinction spectra of colloidal aqueous solutions of AgPRs synthesized five times. **Figure S5.** (a) Enhancement factor of fluorescence obtained from fluorescence excitation spectra for TPP/AgPRs-500, TPP/AgPRs-560, TPP/AgPRs-645, and TPP-ternary (*λ*_em_ = 720 nm) at (i) 435 nm, (ii) 519 nm, (iii) 552 nm, (iv) 596 nm, and (v) 653 nm, respectively. (b) Absorption enhancement at the respective Q-band wavelengths.

## Data Availability

All data generated or analyzed during this study are included in this published article and its supplementary information files.

## Abbreviations

AFM: Atomic force microscopy
AgNO_3_: Silver nitrate
AgPRs: Silver nanoprisms
BEM: Boundary element method
H_2_O_2_: Hydrogen peroxide
IT: Induced transparency
LED: Light-emitting diode
LSP: Localized surface plasmon
NaBH_4_: Sodium tetrahydroborate
NaOH: Sodium hydroxide
NH_3_: Ammonia
PEI: Polyethyleneimine
TEM: Transmission electron microscopy
TPP: Tetraphenylporphyrin
