# Supplementary material for: Combined Use of Anisotropic Silver Nanoprisms with Different Aspect Ratios for Multi-Mode Plasmon-Exciton Coupling
Source: Nanoscale Res Lett. 2020 Jan 16;15:15. doi: 10.1186/s11671-020-3248-8 (PMC6965570; doi:10.1186/s11671-020-3248-8)
Supplement: Supplementary file 1 — Additional file 1: Figure S1. Geometric models of AgPRs with various LSP resonance wavelengths. Figure S2. A Setup for the irradiation of LED light to a colloidal aqueous solution of Ag nanospheres. Figure S3. Normalized extinction and emission spectra of a toluene solution of TPP (1×10^-6^ M). Figure S4. Extinction spectra of colloidal aqueous solutions of AgPRs synthesized five times. Figure S5. (a) Enhancement factor of fluorescence obtained from fluorescence excitation spectra for TPP/AgPRs-500, TPP/AgPRs-560, TPP/AgPRs-645, and TPP-ternary (λem = 720 nm) at (i) 435 nm, (ii) 519 nm, (iii) 552 nm, (iv) 596 nm, and (v) 653 nm, respectively. (b) Absorption enhancement at the respective Q-band wavelengths. [file 11671_2020_3248_MOESM1_ESM.docx]

**Supporting Information**

***Combined Use of Anisotropic Silver Nanoprisms with Different Aspect Ratios for Multi-Mode Plasmon-Exciton Coupling***

Naoto Takeshima,^†^ Kosuke Sugawa,*^,†^ Hironobu Tahara,^‡^ Shota Jin,^†^ Masaki Noguchi,^†^ Yutaro Hayakawa,^†^ Yuhei Yamakawa,^†^ and Joe Otsuki ^†^

^†^Department of Materials and Applied Chemistry, College of Science and Technology, Nihon University, Kanda-Surugadai, Chiyoda-ku, Tokyo 101-8308, Japan

‡Graduate School of Engineering, Nagasaki University, Bunkyo, Nagasaki 852-8521, Japan

*Correspondence e-mail: sugawa.kosuke@nihon-u.ac.jp

**
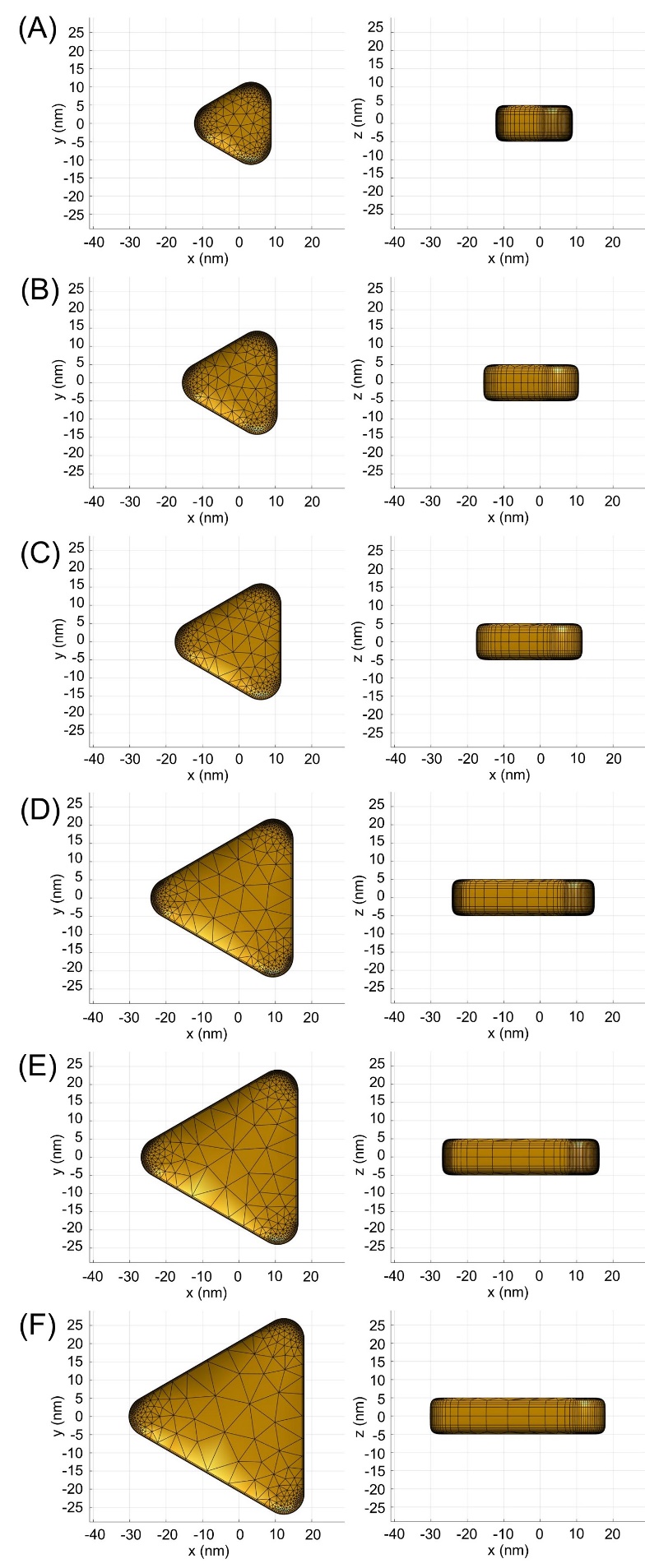
**

**Fig. S1** Geometric models of AgPRs with various LSP resonance wavelengths. (A) AgPRs-500, (B) AgPRs-540, (C) AgPRs-560, (D) AgPRs-625, (E) AgPRs-645 and (F) AgPRs-675.

**
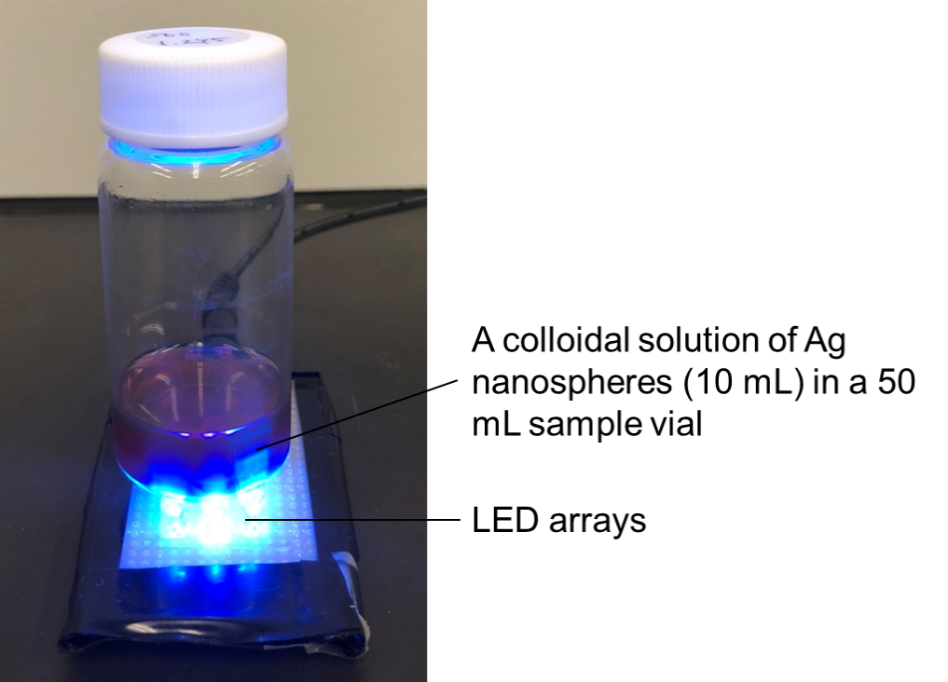
**

**Fig. S2** A Setup for the irradiation of LED light to a colloidal aqueous solution of Ag nanospheres.

**
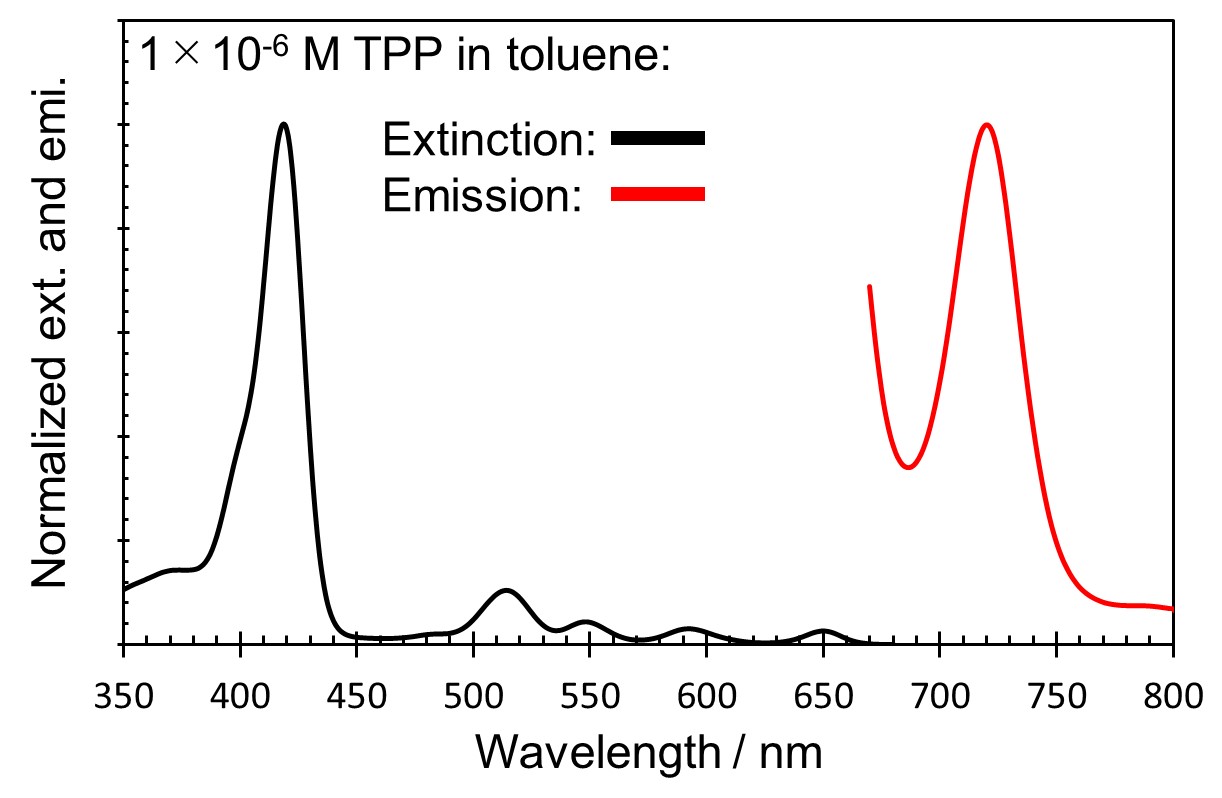
**

**Fig. S3** Normalized extinction and emission spectra of a toluene solution of TPP (1 × 10^-6^ M).

**
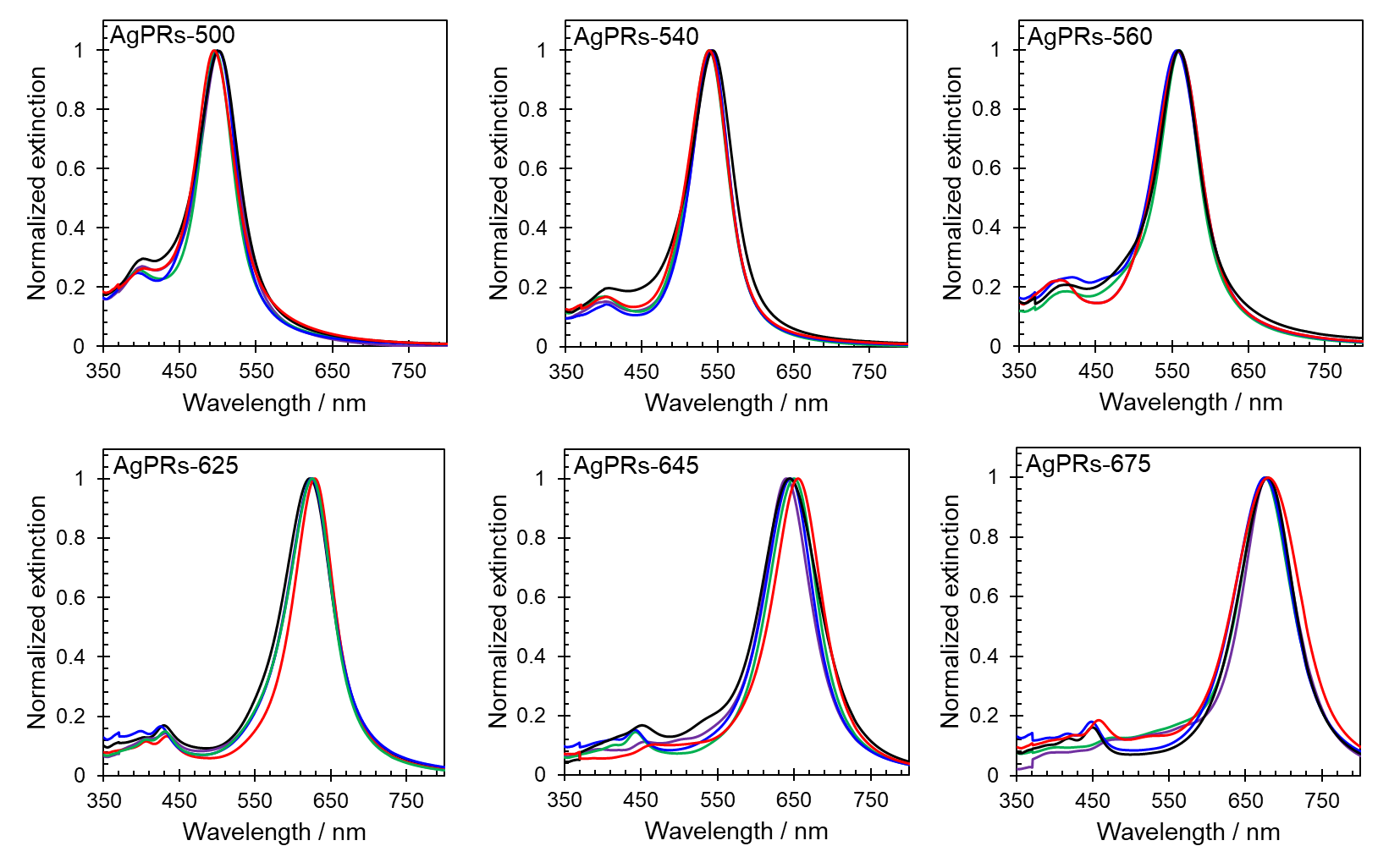
**

**Fig. S4** Extinction spectra for colloidal aqueous solutions of AgPRs each synthesized five times.

**
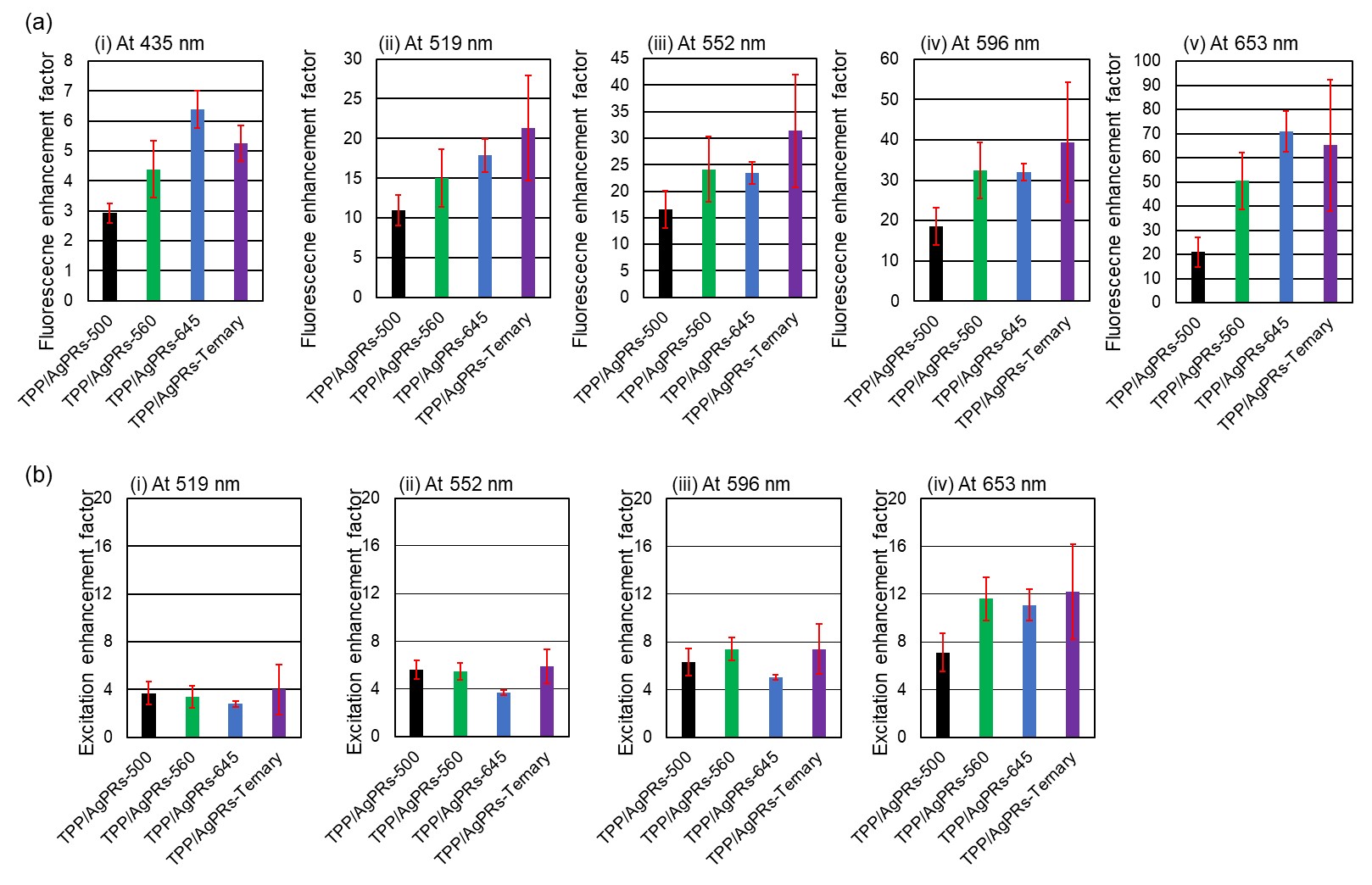
**

**Fig. S5** **a** Enhancement factor of fluorescence obtained from fluorescence excitation spectra for TPP/AgPRs-500, TPP/AgPRs-560, TPP/AgPRs-645, and TPP-ternary (λ_em_ = 720) at (i) 435 nm, (ii) 519 nm, (iii) 552 nm, (iv) 596 nm, and (v) 653 nm (Q-band wavelengths), respectively. **b** Absorption enhancement at the respective Q-band wavelengths.
